# Targeted Therapy for SLE—What Works, What Doesn’t, What’s Next

**DOI:** 10.3390/jcm12093198

**Published:** 2023-04-29

**Authors:** Veronica Venturelli, David Alan Isenberg

**Affiliations:** 1 Rheumatology Unit, Department of Medical Sciences, Università degli Studi di Ferrara, Azienda Ospedaliero-Universitaria S. Anna, 44124 Cona, Italy; 2 Centre for Rheumatology, Department of Medicine, University College London, London WC1E 6JF, UK

**Keywords:** biological therapies, combination therapies, trials, systemic lupus erythematosus, lupus nephritis

## Abstract

For many years, the failure of randomized controlled trials (RCTs) has prevented patients with systemic lupus erythematosus (SLE) from benefiting from biological drugs that have proved to be effective in other rheumatological diseases. Only two biologics are approved for SLE, however they can only be administered to a restricted proportion of patients. Recently, several phase II RCTs have evaluated the efficacy and safety of new biologics in extra-renal SLE and lupus nephritis. Six drug trials have reported encouraging results, with an improvement in multiple clinical and serological outcome measures. The possibility of combining B-cell depletion and anti-BLyS treatment has also been successfully explored.

## 1. Introduction

Systemic lupus erythematosus (SLE) is an autoimmune rheumatic disease characterized by an unpredictable course and highly variable manifestations of differing severity. The main pathogenic feature of the disease is the presence of antibodies directed against autoantigens. The autoantibodies form immune complexes, which precipitate in different tissues and cause progressive organ damage, which has been associated with a higher risk of future mortality, with at least two-fold higher than the general population [1,2].

In the 1950s, the four-year survival for patients with systemic lupus erythematosus was 50% [3]. It is currently in the order of 85% fifteen-year survival [4,5]. This clearly represents a major improvement over the past seventy years, but also implies that the best of the conventional drugs (hydroxychloroquine, prednisolone, immunosuppressives) struggle to contain the disease and clearly some patients with SLE continue to die far too young. Moreover, drug toxicity of conventional therapies has been implicated in the progression of irreversible organ damage [6,7]. In a multi-ethnic study cohort, patients with damage were significantly more likely to have been treated with some immunosuppressants and biologics that are commonly used in clinical practice (azathioprine, mycophenolate, and rituximab) when compared to patients who did not develop damage [8]. This highlights the urgent need to find alternative therapies [9].

The remarkable development of the biologic drugs to treat patients with the autoimmune rheumatic diseases has radically improved the outcome and quality of life for many patients with rheumatoid arthritis, psoriatic arthritis, and ankylosing spondylitis [10]. In stark contrast, relatively few biologic drugs have “made the grade” in the routine management of lupus. B-cell blocking therapeutics currently approved for the use in SLE have proved to be effective in controlling disease activity, reducing the steroid dose [11], and decreasing damage progression in the open-label extension of randomized controlled trials [12]. Unfortunately, not all patients respond to these drugs, with rituximab having a response rate of approximately 70 to 80% [13,14] and belimumab showing at least 50% improvement in approximately half of the patients treated in the OBSErve Study [15]. In addition, some patients with an initial good response to rituximab experience relapse [16], while others develop allergic reactions [17].

However, there are now more encouraging signs of both short and longer-term successes with genuine hope that a number of biologic drugs will become part of the standard repertoire when treating lupus patients. There is an increasing expectation that there will be a much broader selection of biologic drugs to choose from in the next five to ten years.

In this article, we will review the approved biologics for SLE patients, those that were effective in phase II trials and are currently in phase III trials, and future perspectives.

## 2. Conventional Lupus Treatment

**Figure 1:**
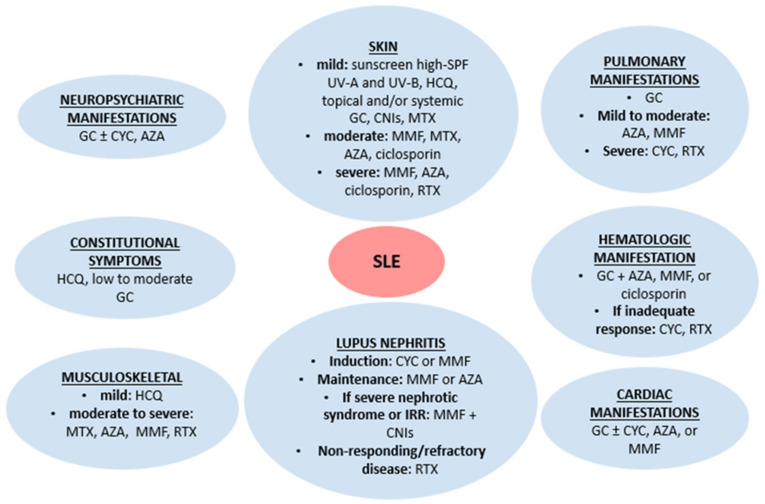
Conventional lupus therapies. AZA: azathioprine. CNIs: calcineurin inhibitors. CYC: cyclophosphamide. GC: glucocorticoids. HCQ: hydroxychloroquine. IRR: incomplete renal response. MMF: mycophenolate mofetil. RTX: rituximab. SLE: systemic lupus erythematosus. SPF: sun protection factor.

Conventional drugs for the treatment of lupus patients have been available for the past twenty to fifty years. Although there is some individual variation (e.g., methotrexate is particularly helpful in inflammatory arthritis), corticosteroids and immunosuppressive drugs are used across an array of common lupus features (Figure 1) [18,19]. Hydroxychloroquine is recommended for all patients, given its beneficial effects on disease control, organ damage, and thrombotic risk [20]. Patients with antiphospholipid syndrome in the context of SLE will invariably require anticoagulation in addition to immunosuppressive therapies.

## 3. Brief Overview of the Immunopathology of SLE and How This Guides the Development of Relevant Biologic Drugs

**Figure 2:**
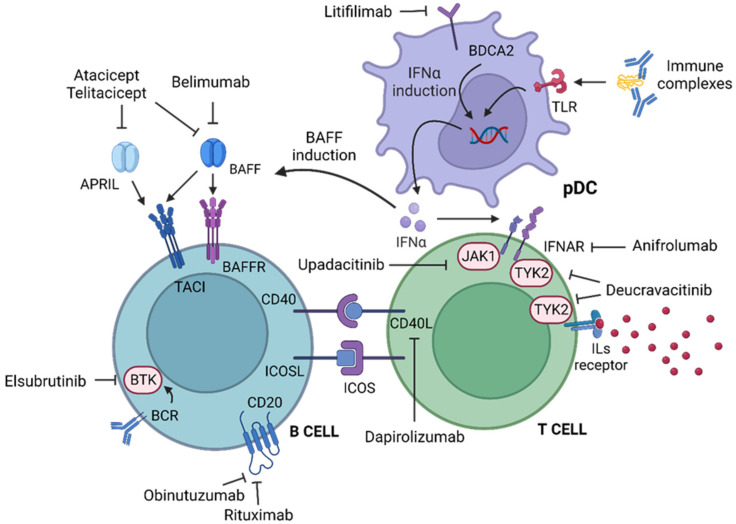
Created with BioRender.com (accessed on 19 April 2023). Molecules and cells involved in the immunopathogenesis of SLE. The mechanism of the drugs discussed in the review is shown. APRIL: a proliferation-inducing ligand. BAFF: B-cell-activating factor. BAFFR: BAFF receptor. BCR: B-cell receptor. BDCA2: blood dendritic cell antigen 2. BTK: Bruton tyrosine protein kinase. CD40L: CD40 ligand. ICOS: inducible T-cell co-stimulator. ICOSL: ICOS ligand. IFNα: interferon α. IFNAR: type 1 interferon receptor. ILs: interleukins. JAK1: Janus kinase 1. pDC: plasmacytoid dendritic cell. TACI: Transmembrane Activator and Calcium-modulator and cyclophilin ligand Interaction. TLR: Toll-like receptor. TYK2: Tyrosine kinase 2.

In the last past twenty years, the growing understanding of the cellular interactions and molecular mechanisms involved in the immunopathogenesis of SLE has allowed the development of several drugs with different targets (Figure 2).

The central event in SLE pathogenesis is the loss of tolerance, which results in the survival of autoreactive B-cells. Different phenomena are thought to contribute to the breakdown of self-tolerance, including the defective clearance of apoptotic debris [21], the inability to degrade neutrophil extracellular traps (NETs) [22], and abnormalities in B-cell receptor (BCR) signaling [23]. After recognizing the antigens on the surface of antigen presenting cells (APCs), BCRs on naïve B-cells initiate a cytoplasmatic signal transduction pathway leading to antigen presentation to T cells [24]. Several molecules are involved in the transduction, notably CD19, CD20, and CD22 [25,26], and many have been reported to be altered or defective in lupus patients and lupus animal models [27,28]. BCR function is also regulated by FcγRIIB, which increases the threshold for B-cell activation [29], and Bruton’s tyrosine kinase (BTK), whose inhibition has improved the manifestations of autoimmune diseases in studies on arthritis and lupus mouse models, suggesting that BTK might be a therapeutic target in SLE [30]. Since rituximab, a chimeric anti-CD20 antibody, first used in SLE patients in 2002 [31], the membrane protein CD20 has increasingly gained attention among researchers because of the possibility of using humanized monoclonal antibodies without the risk of the allergic reactions observed with rituximab. The rationale behind the administration of anti-CD20 antibodies is that their use has been associated with a significant reduction in plasma cell population. This is linked to a reduction in many lupus antibodies including anti-dsDNA, anti-nucleosome and anti-cardiolipin [32,33]. Furthermore, it has been observed that activated CD20+ B lymphocytes are able to activate T cells and can lead to an increased production of cytokines [34].

In SLE, altered expression of costimulatory molecules has also been described. In 2016, Menard et al. analyzed B-cell subsets and T cells in a cohort of SLE patients of African American origin. Among the several molecules studied, they noted the expression of CD40 and CD40 ligand (CD40L), which provide an essential signal for the maturation of B-cells and the isotype switching [35,36]. The results described alterations in the levels of CD40 and CD40L on B and T cells surface, suggesting that this pathway was involved in the more severe course of the disease that is often observed in African American/Afro-Caribbean patients [37]. Closely linked to CD40-CD40L, inducible co-stimulatory molecules (ICOS) and its ligand (ICOSL) are critically involved in the differentiation of B-cells. In SLE mouse models, ICOS can support self-reactive T cell survival [38].

One of the most important factors in the survival of peripheral self-reactive B-cells is the B-cell-activating factor (BAFF), also known as BLyS, a member of the tumor necrosis factor (TNF) family. Thien et al. found that BAFF overexpression rescued self-reactive B-cells from depletion [39]. Moreover, serum BAFF levels have been found to be associated with anti-dsDNA antibody levels and a higher rate of relapse following B-cell depletion therapy with rituximab [40]. In addition to BAFF, another B-cell activating factor, the proliferation-induced ligand APRIL, and TWEAK (TNF-like weak inducer of apoptosis) contribute to the homeostasis of B-cells and have been reported to be increased in SLE patients with lupus nephritis compared to controls [41,42,43].

T cells are thought to play an important role in the pathogenesis of lupus nephritis. In kidney biopsies of SLE patients with renal involvement, T cells are one of the dominant cell populations [44]. In 2020, Zhou et al. found that the administration of tofacitinib, a Janus kinase (JAK) inhibitor, into lupus-prone mouse models led to the prevention of lupus nephritis and to the reduction of the number of CD8+ renal resident memory T cells [45]. With previous evidence of efficacy of JAK inhibitors in lupus animal models [46], this finding supported further investigations in humans.

The interferon (IFN) pathway has also been extensively studied in the context of lupus pathogenesis. In 2005, a French study group demonstrated that IFN-α could induce BAFF mRNA expression in salivary gland epithelial cells of patients with primary Sjogren’s syndrome [47]. This discovery shone a light on the complex interplay between innate and adaptive immune responses; it is thought that self-nucleic acids in autoimmune complexes can increase the secretion of IFN-α by plasmacytoid dendritic cells via Toll-like receptors [48,49]. This rise in IFN-α levels will eventually support B-cells survival and differentiation by inducing BAFF. The role of IFN-α in SLE has been emphasized by several groups, who have reported a dysregulation of interferon gene signatures in different tissues [50], and have shown that type I IFN activity correlated with activity measures [51].

## 4. Development and Current Usage of Biological Drugs Approved for the Treatment of SLE

Two biologic agents, belimumab and anifrolumab, have been approved for use in patients with SLE by the Food and Drug Administration (FDA). The use of rituximab is approved in the UK by the NHS (National Health Service) England and recommended by the European Alliance of Associations for Rheumatology (EULAR) and the American College of Rheumatology (ACR) in severe refractory cases of nephritis. It is also widely used in patients who fail to respond or develop major side effects with conventional immunosuppressants [18].

### 4.1. Rituximab

Rituximab is a chimeric anti-CD20 monoclonal antibody, whose use in SLE was first reported in an open study of six patients in 2002 [31]. It had already been administered successfully to patients with rheumatoid arthritis [52], following the discovery of B-cells infiltrating the synovial membrane of affected joints [53]. In subsequent cohort and open-label studies, both lupus patients with lupus nephritis and those with extra-renal manifestations, notably articular involvement and autoimmune thrombocytopenic purpura, benefited from rituximab or a biosimilar [54,55,56,57]. However, two major trials failed to demonstrate the superiority of rituximab as an add-on therapy over the standard of care in achieving clinical response in lupus nephritis and extra-renal disease [58,59]. The Exploratory Phase II/III SLE Evaluation of Rituximab (EXPLORER) trial was a randomized, double-blind, placebo-controlled trial based in North America studying the efficacy and safety of rituximab in moderate to severe extra-renal SLE manifestations. The primary endpoint was defined as the percentage of clinical responses measured by the British Isles Lupus Assessment Group index (BILAG) at week 52. The results showed no difference in the proportion of patients achieving a complete or partial response between the rituximab group and the placebo group [58].

With regard to proliferative lupus nephritis (LN), a phase III randomized controlled trial (RCT) of rituximab as an add-on therapy (LUNAR trial) failed to demonstrate superiority over mycophenolate and corticosteroids, compared to placebo in achieving complete or partial renal response [59]. The possible reasons why these trials failed have been carefully considered [60]. It is thought that some clinical features might have been misclassified as activity, leading to biased results that did not show any improvement because the features were actually due to damage. The extreme heterogeneity of SLE among different groups of patients might be implicated. Indeed, secondary analysis of the population enrolled in the EXPLORER trial highlighted the potential benefit of rituximab in African American patients [58]. Other reasons why the trials were not able to detect any significant benefit of rituximab may be the substantial background medication (steroids and conventional immunosuppressants) prescribed for the population in the trial and the inadequate sensitivity of the outcome measures. In 2016, the BAFF surge observed in some patients after the administration of rituximab has been hypothesized to be linked to the unsuccessful RCTs of rituximab in lupus [61]. This observation has led to further trials studying the efficacy of treatment schemes combining rituximab and belimumab, that will be discussed later.

Despite the disappointing clinical results of the RCTs, in 2019 EULAR recommended rituximab as an induction regimen for patients with extra-renal disease after the failure of multiple therapeutic options, or in case of contraindications to conventional immunosuppressants [18]. It also recommended rituximab alone or added to mycophenolate mofetil or cyclophosphamide in non-responding patients with lupus nephritis [62].

In 2022, Chen et al. investigated the potential role of rituximab as maintenance therapy in a multicenter prospective observational cohort study. Eighty-two patients were enrolled and were all treated with one course of rituximab to induce remission. Subsequently, those who responded clinically within 6 months were divided into two different groups, those receiving the maintenance therapy with rituximab in a single dose or conventional immunosuppressants. The relapse rate in the group treated with rituximab was statistically significantly reduced compared to the group on immunosuppressive agents. This suggests that rituximab might be a valuable treatment to maintain a sustained remission in SLE [63]. The doses and frequency of rituximab in the maintenance setting are not yet well defined. A randomized controlled trial (NCT04127747) has recently focused on two different administration schemes to determine whether an individualized scheme based on CD19+ B-cells, dsDNA, and C3 can control the disease better than a standard one. The group on standard dose will receive an infusion of rituximab 500 mg on the first day, on the 6th, 12th, 18th and 24th month, while the patients on individualized dose will receive rituximab 500 mg on the first day and then will be followed-up every 3 months and will receive another infusion of rituximab 500 mg, if CD19+ B-cell count ≥1%, or anti-dsDNA antibodies titre increased, or complement C3 level decreased. The results are awaited.

### 4.2. Belimumab

Belimumab (Benlysta) is a human monoclonal antibody that neutralizes the soluble form of BLyS. It was the first biological drug approved by the FDA specifically for the treatment of SLE. Advances in biotechnology turned out to be vital for the development of belimumab [64]. In the 1990s, an analysis of human neutrophil-derived cDNA led to the identification of the gene encoding BLyS. After the observation that BLyS-transgenic mice had developed clinical and serological features similar to lupus [65] in 2001, the levels of BAFF were measured in lupus patients and were found to be elevated compared to healthy controls. In this study, BAFF correlated with IgG levels and with anti-dsDNA antibodies [66]. This discovery supported the idea that BAFF could be a candidate therapeutic target in SLE. Belimumab was developed using the technology of human single chain variable fragment (scFv) phage display, which allowed the selection of a limited number of scFvs, those with highest binding and inhibitory activity against BLyS. Then, the selected scFvs underwent a phase of conversion to obtain full-length immunoglobulins and were further processed. The result was a fully human monoclonal antibody, eventually named “belimumab” [67].

In 2009, the results of a phase II trial of belimumab were published. The study did not meet its co-primary endpoints of change using the Safety of Estrogens in Lupus National Assessment-Systemic Lupus Erythematosus Disease Activity Index (SELENA-SLEDAI) and time to first flare. It appeared that belimumab was not better than placebo in controlling disease activity in lupus patients. However, a secondary analysis suggested that it could stabilize the disease [68].

More encouragingly, two phase III trials (BLISS-52 and BLISS-76) reported the superiority of belimumab over placebo in SLE patients (especially those with high anti-dsDNA antibodies and low C3 levels) without severe renal or central nervous system (CNS) involvement. Belimumab was added to the background therapy with conventional immunosuppressants and steroids. In both BLISS-52 and BLISS-76, two different doses of intravenous belimumab were tested (1 mg/kg and 10 mg/kg). BLISS-52 reported higher proportions of response rate, measured with the SRI-4 (Systemic lupus erythematosus Response Index), in belimumab-treated groups regardless of the dose (51% for 1 mg/kg, 58% for 10 mg/kg, 48% for placebo). In BLISS-76, the benefit was significant only in patients given 10 mg/kg of belimumab (43.2% of SRI responders versus 33.5% in the placebo group, *p* = 0.017), whereas the risk of severe SELENA-SLEDAI flare over 76 weeks was significantly reduced only in those given 1 mg/kg of belimumab (18.5% versus 26.5% in placebo group, *p* = 0.023). In both studies, no increase in the rates of adverse events was noted in the groups on active treatment [69,70]. In BLISS-52 and BLISS-76 patients of Black/African American ancestry and Asian ethnicity were underrepresented, accounting for approximately 25% and less than 5% in BLISS-76, respectively [70]. Further studies were conducted to assess the efficacy of belimumab in these minorities. In Asian patients enrolled in the BLISS-NEA trial, a significant improvement in disease activity and a reduction of the cumulative steroid dose in patients given belimumab was noted [71]. In contrast, the EMBRACE study in African Americans failed to meet the primary endpoint of superiority of belimumab over standard of care, although a higher but insignificant response rate was observed in patients on active treatment compared to placebo [72]. Intravenous belimumab at the dose of 10 mg/kg every 4 weeks as an add-on therapy over standard of care was also assessed in pediatric patients aged >4 years, with results similar to those reported in RCTs on adults [73].

In 2011, the US FDA and the European Medicines Agency (EMA) approved intravenous belimumab at the dose of 10 mg/kg for the treatment of active SLE in adult patients with positive serology who were receiving standard of care. In the UK, the National Institute for Health and Care Excellence recommended the use of belimumab for SLE only in 2016 under strict restrictions (e.g., the patients had to have a SLEDAI-2K score of >10 and could only have skin and/or joint disease). Two years later, the FDA approval was extended to pediatric patients aged five years and above. However, the route of administration impacts heavily on the costs the Health Service has to bear though, by forcing patients to go to the hospital for the infusion regularly, uncertainty about patients compliance is abolished. In terms of treatment adherence, this is beneficial. The BLISS-SC trial demonstrated the equivalent safety and efficacy of subcutaneous belimumab at a fixed dose of 200 mg administered once a week when given in addition to standard of care. The beneficial effect of belimumab was clear early, with significant higher response rates detected at the end of the second month of treatment, which was maintained throughout the study [74].

Although severe active lupus nephritis was an exclusion criterion in BLISS-52 and BLISS-76, a post-hoc analysis underlined the potential improvement in renal parameters in those patients enrolled with mild to moderate renal involvement who were assigned randomly to belimumab treatment. A higher remission rate, shorter time to achieve the remission status, lower risk of flare, and more marked reduction in proteinuria than patients on standard therapy alone was noted [75]. Based on these promising data, The Belimumab International Study in Lupus Nephritis (BLISS-LN) assessed the addition of intravenous belimumab to standard therapy with cyclophosphamide or mycophenolate mofetil. A significantly greater proportion of belimumab-treated patients compared to placebo-treated patients achieved the primary efficacy renal response at week 104, 43%, and 32%, respectively (*p* = 0.03). The primary efficacy renal response was determined by a combination of urine protein creatinine ratio ≤0.7, an estimated glomerular filtration rate no worse than 20% compared to the value recorded before the flare or at least 60 mL/min/1.73 m^2^, and without the need for rescue therapy [76]. These results led to the FDA approving belimumab for lupus nephritis. However, it must be acknowledged that, despite the approval of belimumab in LN, there are some reports of patients who developed renal involvement while taking belimumab [77,78,79].

The Belimumab Assessment of Safety in SLE (BASE) trial showed, in 2019, an increased risk of psychiatric issues in patients on belimumab [80], leading the Medicines and Healthcare products Regulatory Agency to express concern about the safety profile of the drug. However, a subsequent meta-analysis of eleven randomized controlled trials (eight thousand eight hundred and twenty-four patients) did not confirm the excess risk of psychiatric events [81].

Currently, belimumab is approved for extra-renal and renal SLE in both adult and pediatric SLE patients with high disease activity and positive serology (high anti-dsDNA and low complement). Its use in severe active central nervous system involvement in SLE is not recommended, although not on the basis of any compelling data.

### 4.3. Anifrolumab

The increasing amount of evidence about the role of IFN-α in lupus, reviewed by Ramaswamy et al. [82], confirmed the efficacy and safety of anifrolumab, a fully human antibody IgG1k against the type I IFN-α/β/ω receptor, in randomized controlled trials. In the phase IIb MUSE (MEDI-546 in Uncontrolled Systemic lupus Erythematosus) trial, a significantly greater proportion of patients treated with anifrolumab plus standard therapy achieved an SRI-4 response with sustained reduction of steroids at week 24 (34.3% for the group treated with anifrolumab 300 mg, 28.8% for 1000 mg) compared to placebo-treated patients (17.6%). When stratifying the patients according to the IFN signature, a greater effect was found in those with a high type I IFN signature (36% for anifrolumab 300 mg, 28.2% for anifrolumab 1000 mg, 13.2% for placebo patients). This result suggested the possibility of utilizing a more precision mode of treatment for some SLE patients. A higher incidence of herpes zoster was reported in patients given anifrolumab [83].

The results of the first phase III RCT, TULIP-1 (Treatment of the Uncontrolled Lupus via the Interferon Pathway-1), of anifrolumab in extra-renal SLE were published in 2019. Two doses of anifrolumab were evaluated (300 mg and 150 mg) over a 48-week period, followed by 4 weeks of follow-up. At week 52 there was no difference in the SRI-4 response between anifrolumab and the placebo group (primary endpoint). However, the BICLA (BILAG-based Composite Lupus Assessment) responses were higher in anifrolumab-treated patients [84]. The authors of the study suspected that the primary endpoint was not reached because the design was too strict and because the SRI was not able to detect partial improvements. As with belimumab RCTs, a careful choice of the outcome measure was vital. Interestingly, a post-hoc analysis of BICLA and SRI-4 discordant responder status highlighted that, in TULIP-1, the subgroup of placebo-treated patients, who achieved an SRI-4 response but not BICLA response, had lower baseline joint count scores and took higher steroid dose compared to anifrolumab group. These factors might have prevented the authors from detecting significant differences between treatment groups [85]. In the second phase III RCT, TULIP-2, SRI-4 was replaced by BICLA as the primary outcome measure. Patients with active severe lupus nephritis or neuropsychiatric SLE were again excluded from the study. TULIP-2 demonstrated the superiority of intravenous 300 mg of anifrolumab administered monthly in active moderate to severe SLE over placebo. A greater proportion of patients treated with anifrolumab achieved a BICLA response compared to placebo at week 52 (47.8% versus 31.5%, *p* = 0.001). Ironically, the SRI-4, a secondary outcome measure in this study, was also met. When the population was stratified according to the interferon signature, only in the subpopulation characterized by a high interferon signature was a significantly higher percentage of BICLA-responders noted (48.0% versus 30.7%, *p* = 0.002) [86].

In 2021, the FDA approved the use of anifrolumab over standard treatments in adult lupus patients with moderate to severe manifestations without severe active renal or neuropsychiatric involvement. Currently, a 116-week phase 3 randomized controlled trial (IRIS) is recruiting lupus patients to evaluate the efficacy and safety of intravenous infusion of anifrolumab plus standard of care (mycophenolate mofetil and corticosteroids) in active class III or IV lupus nephritis (NCT05138133).

## 5. Reviewing Those Drugs That Have Achieved Phase II Drug Trial Endpoints and Are in or about to Start Phase III Trials

### 5.1. Obinutuzumab

Obinutuzumab is a fully humanized anti-CD20 monoclonal antibody which causes a more complete B-cell depletion than rituximab [87].

A phase II trial (NOBILITY, Table 1) [88] of obinutuzumab added to mycophenolate and steroids studied 242 SLE patients aged 18–75 years with histologic evidence of class III or IV lupus nephritis, a urine protein to creatinine ratio (UPCR) >1 and an estimated glomerular filtration rate ≥30 mL/min/1.73 m^2^. Sixty-three patients were randomly assigned to the obinutuzumab group and followed until week 104. Obinutuzumab 1000 mg or placebo was administered intravenously on day 1, week 2, 24, and 26. The primary endpoint, evaluated at week 52, was the proportion of complete renal responses (CRR), defined by UPCR <0.5, serum creatinine within normal range, absence of worsening of serum creatinine by >15%, and <10 red blood cells per high-power field in urinary sediment without red blood cells casts. The trial reached its primary objective, with a greater proportion of patients in the obinutuzumab group who achieved CRR at week 52 compared to the placebo, 35% and 23%, respectively (*p* = 0.115 with a prespecified alpha level of 0.2). In addition to CRR, partial renal responses (PRR) were also considered as a component of the overall renal responses (ORR), including both CRR and PRR. The requirements to achieve PRR were the UPCR reduced by at least 50% from baseline down to values <1 or <3 if the baseline UPCR was ≥3, a worsening in the serum creatinine of less than 15%, <10 red blood cells per high-power field in the urinary sediment or less than 50% increase from baseline. At week 52, a higher proportion of patients in the obinutuzumab group achieved ORR than in the placebo group (approximately 55% versus around 35%, *p* < 0.05). Notably, this advantage was maintained throughout the study (*p* < 0.01 at week 104), despite the last dose of the drug having been administered at week 26. The benefit of obinutuzumab was particularly evident in class IV lupus nephritis and in patients with baseline UPCR ≥ 3. No improvement in outcome was observed in those patients with LN class V.

Although the proportion of patients with B-cell depletion (CD19+ ≤5 cells/µL) was similar between groups at week 104, the CD19+ B-cells in patients taking obinutuzumab were depleted more rapidly than in patients given the placebo. Indeed, at week 2, the levels of CD19+ cells were <5 cells/µL in 98% of patients treated with obinutuzumab, compared to the placebo group in which only 2% of patients achieved B-cell depletion. Treatment with obinutuzumab was associated with low IgM levels at week 104 (33% of patients versus 8% in the placebo group) and with early significant improvement in anti-dsDNA antibodies and complement levels. Considering safety, the rate of serious infections in the obinutuzumab group (8%) was not higher than in the placebo group (18%). Obinutuzumab-treated patients had a slightly higher incidence of non-severe infusion-related reactions (notably headache, tachycardia, nausea, and hypertension) than placebo-treated patients, 16% and 10%, respectively.

These data have led to the development of a phase III trial (REGENCY) of obinutuzumab in class III or IV lupus nephritis. For this study, investigators are asked to recruit patients aged 18–75, with an estimated enrollment of two hundred fifty-two patients, who will be randomized into three different treatment groups. Those in group 1 will receive 1000 mg of intravenous obinutuzumab at baseline and weeks 2, 24, 26, 50, and 52, in addition to mycophenolate mofetil and oral prednisone; patients in group 2 will receive the same treatment except for the infusion at week 50, that will be replaced by placebo, and those in group 3 will receive placebo infusions plus mycophenolate mofetil and oral steroids. The primary endpoint will be the percentage of complete renal responses at week 76.

Another phase III trial (OBILUP, NCT04702256) will explore the efficacy of obinutuzumab in proliferative lupus nephritis (class III or IV). The OBILUP study is a randomized, open-label, controlled, non-inferiority trial investigating the association of obinutuzumab and mycophenolate mofetil compared to mycophenolate mofetil plus steroids in achieving complete renal response in adolescents (aged 14 years and above) and adults. The patients in the obinutuzumab group will not be given oral steroids unless they have extrarenal involvement. In those with extrarenal manifestations, the steroid dose will be kept under 10 mg/day at any time, <7.5 mg/day after 6 months and <5 mg/day after 9 months.

The potential role of obinutuzumab in extra-renal SLE will be evaluated in a phase III trial (ALLEGORY, NCT04963296) including only patients with anti-nuclear antibody (ANA) ≥ 1:80, or elevated anti-dsDNA and/or anti-Sm antibodies, hypocomplementemia C3 and/or C4 and/or low CH50. Other key inclusion criteria will be high disease activity at screening, defined according to the BILAG-2004, the Systemic Lupus Erythematosus Disease Activity Index 2000 (SLEDAI-2K) and Physician’s Global Assessment (PGA). The therapy will be administered with the same scheme used in the NOBILITY trial. The primary objective will be the proportion of SRI-4 responders at week 52. The response rates according to SRI-6, SRI-8 and BICLA will be also assessed as secondary endpoints at week 52.

### 5.2. Dapirolizumab

Dapirolizumab is a polyethylene glycol (PEG)-conjugated antigen-binding fragment (Fab’) targeting CD40L. It lacks the functional fragment crystallizable domain, that has been implicated in the thromboembolic events reported with previous anti-CD40 drugs [89]. In early clinical trials, dapirolizumab was well tolerated and the rate of adverse events was similar between dapirolizumab and placebo groups [90,91].

A phase IIb, randomized, double-blind, placebo-controlled trial (Table 2) [92] aimed to establish a dose-response relationship and investigated the safety and efficacy of dapirolizumab administered intravenously in addition to standard of care in moderate to severe SLE. Patients with a new diagnosis of lupus nephritis (class III or IV), worsening pre-existing LN, and neuropsychiatric SLE were excluded from the trial. One hundred eighty-two patients, aged 18 years or above, were enrolled and randomly assigned to one of four groups receiving intravenous dapirolizumab at three different doses (6 mg/kg, 24 mg/kg, or 45 mg/kg) or intravenous placebo monthly up to week 20. All patients, then, underwent an observational period, lasting 24 weeks. One hundred sixty-eight(90.1%) patients completed the study. The trial was unable to identify a dose-response relationship (its primary endpoint) even when a stratification for baseline steroid dose was performed. For the primary objective the responses were determined using BICLA at week 24. An overall greater improvement in SRI-4, BICLA, SLEDAI-2K, PGA, BILAG, and Cutaneous Lupus Erythematosus Disease Area and Severity Index–Activity (CLASI-A) was observed throughout the study in dapirulizumab-treated patients when compared to placebo-treated patients, together with a greater reduction in the levels of anti-dsDNA and antiphospholipid antibodies and a rise in C3 and C4. There were also fewer flares in the group on dapirolizumab compared to the placebo (5 versus 7). After the discontinuation of the treatment at week 24 required by the study protocol, auto-antibodies and complement components tended to return to pre-treatment values. During this period of observation, a stabilization of BILAG, SLEDAI, and PGA was observed, where there was a reduction of the BICLA and SRI-4 response rates, probably due to the use of rescue therapy. Unlike earlier anti-CD40L monoclonal antibodies, dapirolizumab was not associated with a higher risk of thromboembolic events compared to placebo. Its safety profile was considered acceptable; severe treatment-associated adverse events (AEs) were similar in the treatment groups.

Importantly, this phase II study did not have as its primary endpoint the response rate. Thus, the failure to meet the primary objective cannot be considered as evidence of inefficacy. In contrast, it was felt that the overall improvement in the secondary endpoint supported further investigation and the subsequent design of a phase III, randomized, controlled trial (PHOENYCS GO), which will assess dapirolizumab in lupus patients with moderate or severe disease in spite of standard of care. Approximately four hundred fifty patients, aged 16 and above, will be enrolled. Those with neuropsychiatric manifestation or renal involvement causing estimated glomerular filtration rate <30 mL/min/1.73 m^2^, serum creatinine >2.5 mg/dL, proteinuria >3 g/day, or protein to creatinine ratio >340 mg/mmol, will be excluded. The primary outcome measure will be the proportion of BICLA responders at week 48. The study will have multiple secondary endpoints, notably the proportion of patients achieving SRI-4 response at week 48, BICLA response at week 12 and at week 24, and Lupus Low Disease Activity State (LLDAS) ≥50% of post-baseline visit. Other secondary outcome measures will be the rate of BILAG flare until the end of week 48, BILAG improvement without worsening at week 48, the time to first BILAG flare, the change in PGA from baseline at week 48, and the rate of adverse events from baseline up to week 54 (NCT04294667). The safety and tolerability of dapirolizumab will be also assessed in an ongoing phase III open label study (NCT04976322) evaluating the incidence of treatment-emergent adverse events up to week 110 in seven hundred sixty patients aged ≥16 years. The secondary endpoints will be the efficacy measures (proportion of BILAG flares at different time points, achievement of LLDAS, BICLA responses).

### 5.3. Deucravacitinib

Deucravacitinib is a highly selective Tyrosine Kinase 2 (TYK2) inhibitor whose activity is due to the binding to a regulatory site which results in allosteric inhibition [93].

The phase II trial (PAISLEY, Table 3) [94] of deucravacitinib in SLE evaluated the efficacy and safety of three different dosages (3 mg twice daily, 6 mg twice daily, or 12 mg once daily) in active SLE with at least one BILAG-2004 grade A or at least 2 BILAG-2004 grade B from the musculoskeletal or mucocutaneous domain and SLEDAI-2K ≥6. Three hundred sixty-three patients aged 18–75 years were included, of whom ninety-one received the lowest dose of the drug, ninety-three received 6 mg twice daily, and eighty-nine received 12 mg once daily. The remaining patients received placebo tablets. All patients were taking standard of care treatment. Deucravacitinib was administered orally for 48 weeks. Patients with severe, active lupus nephritis, neuropsychiatric SLE, recent herpes zoster, herpes simplex or influenza infection or history of disseminated or complicated herpes zoster were excluded. The primary endpoint of the proportion of SRI-4 responders at week 32 was met. In the patients taking 3 mg twice daily of deucravacitinib, there was a significantly higher percentage of SRI-4 responders than in the placebo group (58.2% versus 34.4%, *p* <0.001). A significantly higher proportion of SRI-4 responses (49.5%) was also noted among those taking 6 mg twice daily dose when compared with placebo (*p* = 0.02). No significant difference was observed between patients on deucravacitinib 12 mg daily and the placebo group.

With regard to the secondary endpoints evaluated at week 48, only the group taking deucravacitinib 3 mg twice daily differed significantly when compared with placebo. In fact, it showed higher proportions in SRI-4 responses (57.1% versus 34.4%, *p* < 0.001), BICLA responses (47.3% versus 25.6%, *p* = 0.001), CLASI-50 responses (69.6% versus 16.7%, *p* < 0.001), LLDAS (36.3% versus 13.3%, *p* < 0.001), and a significantly greater mean change from baseline in the joint count (–8.9 versus –7.6, *p* = 0.001). The patients taking deucravacitinib also showed an increasing improvement in the levels of anti-dsDNA antibodies and C3 and C4 levels throughout the study and a dramatic reduction in the IFN gene expression from week 4 was noted.

In terms of safety, AEs were mainly mild or moderate infections, with upper respiratory tract infections and nasopharyngitis being the most common. Encouragingly, the incidence of herpes zoster infection, a well-known complication of the treatment with JAK inhibitors [95], was similar between the deucravacitinib and the placebo groups. However, there was a higher frequency of cutaneous adverse events (acne and rash) in deucravacitinib-treated patients compared with placebo (16.5% in the group taking 3 mg twice daily of deucravacitinib, 34.4% in the group taking 6 mg twice daily, 33.7% in the group taking 12 mg once daily, 13.3% in the placebo group).

The promising results of the PAISLEY trial have led to the design of two-phase III trials (POETYC SLE-1—NCT05617677- and POETYC SLE-2 -NCT05620407-), which will evaluate the SRI-4 response rate at week 52 in patients with extra-renal SLE treated with deucravacitinib in addition to standard of care. These studies will have a long-term follow-up to detect adverse events up to week 156.

### 5.4. Litifilimab

Litifilimab is a humanized monoclonal antibody that targets the surface receptor blood dendritic cell antigen 2 (BDCA2). BDCA2 is expressed on plasmacytoid dendritic cells [96] and has been linked to the production of interferon α [97].

Litifilimab was evaluated in a two-part, phase II, multicenter, randomized, controlled trial (LILAC, Study to Evaluate the Efficacy of BIIB059 in Subjects with Systemic Lupus Erythematosus and Active Skin Manifestations and in Subjects with Cutaneous Lupus Erythematosus, Table 4) [98]. Here, we will only review the results of part A of the study. Part B involved patients with cutaneous lupus erythematosus, some of whom did not have systemic involvement.

Part A of the LILIAC study enrolled adults with a positive antinuclear antibody test ≥1:80 and/or anti-dsDNA antibodies ≥30 UI/mL, active skin disease defined as a score of at least 8 on the CLASI-A, a score of at least 4 on the SLEDAI-2K. According to the second version of the protocol, a minimum score on the CLASI-A was no longer required, but patients had to have at least one active skin lesion and at least four tender and swollen joints in the sites evaluated with the twenty-eight joint assessment to be considered eligible. The study met its primary endpoint and demonstrated the superiority of litifilimab 450 mg over standard of care in controlling joint involvement in SLE. The least-squares mean (LSM) difference in the number of active joints between litifilimab and placebo at week 24 was −3.4 (*p* = 0.04). When compared to the placebo, the group treated with litifilimab showed a significantly higher proportion of patients who experienced a decrease of at least 7 points on CLASI-A score (56% versus 34%, with LSM difference [95% confidence interval -CI-]: 21.6 [0.1 to 43.1]), a greater number of SRI-4 responders (56% versus 29%, with LSM difference [95% CI]: 26.4 [9.5 to 43.2]), and a greater absolute change in SLEDAI-2K (LSM –4.4 ± 0.5 versus –2.6 ± 0.5, with LSM difference [95% CI]: –1.7 [–3.0 to –0.5]). All these secondary outcome measures were evaluated at week 24. The serologic analysis of patients in the litifilimab arm did not identify any improvement in the anti-dsDNA antibodies, C3, C4, or immunoglobulins levels. The placebo group reported more AEs than the litifilimab group (68% versus 59%). AEs were mostly mild to moderate, with only 5% of litifilimab-treated patients and 11% of placebo-treated patients experiencing serious AEs. The most common AEs in the litifilimab group were diarrhea, nasopharyngitis, and urinary tract infections, each occurring in seven patients (5%).

Based on the promising results of the LILIAC trial, two phase III trials (TOPAZ-1 -NCT04895241- and TOPAZ-2—NCT04961567-) of low and high doses of litifilimab as an add-on therapy over standard of care in lupus patients have been designed and are ongoing. Both will measure the proportion of SRI-4 responders at week 24 as primary objective measure. Multiple secondary endpoints will be considered, including the joint count assessment, the change in CLASI-A score, and the reduction of steroid dose. The patients who will complete the TOPAZ-1 and TOPAZ-2 trials will be offered the possibility of entering the extension study (EMERALD, NCT05352919), lasting 180 weeks. The primary endpoint of the EMERALD trial will be the safety profile of litifilimab. The proportion of patients experiencing treatment-emergent and serious AEs, and the long-term impact of litifilimab on several efficacy outcome measures will be evaluated.

Another phase II/III trial of litifilimab (AMETHYST, NCT05531565) is currently re-cruiting patients with cutaneous SLE with or without systemic involvement who did not respond to antimalarial treatment. The primary endpoint will be the control of skin manifestations (achievement of Cutaneous Lupus Activity of Physician’s Global Assessment-Revised (CLA-IGA-R) Erythema Score of 0 or 1 and CLASI-70 responses).

### 5.5. Atacicept

Atacicept is a recombinant fusion protein constituted by the extracellular domain of TACI (Transmembrane Activator and Calcium-modulator and cyclophilin ligand Interaction) receptor and a modified portion of human IgG [99]. TACI is the receptor of two B- cell activating factors, BAFF and APRIL, and is normally expressed on B-cells [100]. Thus, atacicept binds BAFF and APRIL preventing them from activating the downstream signaling pathway. The two major phase II trials that investigated atacicept in extra-renal SLE did not meet their primary endpoints [101,102] and the phase II RCT of atacicept plus mycophenolate in lupus nephritis was terminated due to the occurrence of serious infections thought to be linked to hypogammaglobulinemia initially believed to be due to the atacicept, but in fact the concomitant mycophenolate is the more likely culprit [103]. Since the phase II trial of atacicept in lupus nephritis was interrupted after the enrollment of just six patients, the efficacy of the drug has not been adequately tested [103]. Recently Vera Therapeutics has acquired atacicept (from Merk Serono) and a phase III trial (COMPASS, NCT05609812) of atacicept in adult lupus patients with biopsy-proven active lupus nephritis is ongoing. It will last for 104 weeks, followed by 52 weeks of open-label treatment.

Here, we will summarize the design and main results of the phase II trials of atacicept in SLE (APRIL-SLE [101] and ADDRESS II [102], Table 5). The APRIL-SLE trial [101] was a 52-week, randomized, placebo-controlled, phase II/III trial investigating two different doses of atacicept (75 mg and 150 mg) after patients who had flared had been admitted into remission with steroids. The atacicept/placebo was given in addition to standard of care to prevent flares in patients with extra-renal moderate-to-severe SLE. The therapy was administered twice weekly for 4 weeks, then weekly up to week 48. Four hundred sixty-one patients aged 16 years or above were randomized. One hundred forty-five patients were administered atacicept 150 mg, one hundred fifty-nine were administered atacicept 75 mg and one hundred fifty-seven were assigned to the placebo group. The atacicept 150 mg arm was terminated before week 52 because of two fatal infections reported in this group (leptospirosis and pneumococcal pneumonia). This was an odd and unfortunate decision especially as other trials were not stopped in spite of many more deaths (e.g., the voclosporin phase II trial [104]). These events were not associated with hypogammaglobulinemia and the rate of serious infections in the APRIL-SLE study (6.9%) was not higher than the one reported in previous studies of belimumab [70] or rituximab [58], approximately 7% and 9.5%, respectively. With regard to the primary endpoint, atacicept 75 mg did not show superiority over the placebo in preventing BILAG A or B flares at week 52. However, a post-hoc analysis highlighted the potential benefit that atacicept 150 mg might have on flare prevention and on prolonging the time to first flare. When compared to the placebo, the flare rate in this group was significantly lower (37% versus 54%, *p* = 0.002) and the first flare was significantly delayed (Hazard Ratio, HR, 0.56, *p* = 0.009).

Another phase II, randomized, placebo-controlled trial (ADDRESS II) [102] explored the potential role of two different doses of subcutaneous atacicept (75 mg or 150 mg) in adults with moderate to severe SLE (SLEDAI-2K ≥6) receiving standard therapy. Patients with severe lupus nephritis defined by UPCR >2.0 mg/mg and/or estimated glomerular filtration rate <40 mL/minute/1.73 m^2^) or with central nervous system manifestation were excluded. A total of three hundred and six patients were enrolled, of whom one hundred and two were assigned randomly to atacicept 75 mg once weekly, one hundred and four to atacicept 150 mg once weekly, and one hundred to the placebo group. The primary efficacy endpoint (SRI-4 responses at week 24) was not met. However, the exploratory analyses considering day 1 as baseline showed a significantly higher proportion of SRI-4 responders at week 24 in both atacicept groups when compared with the placebo group. Furthermore, the subgroup analyses highlighted a significantly higher SRI-4 response rate in atacicept-treated patients when only those with serologically active disease were considered (61.2% of those receiving atacicept 75 mg and 61.5% of those receiving atacicept 150 mg, with p equal to 0.003 and 0.002, respectively). In the subgroup treated with 150 mg of atacicept and with baseline high disease activity (HDA), defined by SLEDAI-2K score ≥10, 62.7% of patients achieved SRI-4 response at week 24. This percentage was significantly higher compared to placebo-treated patients with baseline HDA (42.3%, *p* = 0.029). A higher proportion of patients taking atacicept 150 mg also achieved SRI-6 response at week 24 compared to placebo (54.9% versus 28.8%, *p* = 0.005). The group taking atacicept 75 mg had less severe BILAG A flares than placebo, regardless of the severity of the disease at baseline (Hazard Ratio [HR] 0.24, *p* = 0.019). When only the subgroup with HDA was considered, a significant difference between the percentages of patients free from severe BILAG A flares was detected between the groups on atacicept 75 mg (HR 0.08, *p* = 0.002) or 150 mg (HR 0.32, *p* = 0.038) and the placebo group. Thus, it seemed that atacicept prevented flares, especially in patients with high disease activity.

In terms of the effect on biomarkers, atacicept was associated with an increase in the levels of complement components (C3 and C4) and with a reduction in the levels of anti-dsDNA antibodies. Significantly higher rates of upper respiratory tract infections (9.8% with atacicept 75 mg and 12.5% with atacicept 150 mg) and diarrhea (6.9% with atacicept 75 mg and 11.5% with atacicept 150 mg) were reported in patients on active treatment compared to the placebo group, in which 3.0% had an upper respiratory tract infection and 5.0% had diarrhea. Overall, the safety profile of atacicept was considered acceptable by the authors.

The long-term extension (LTE) study [105] confirmed the results of the ADDRESS II trial, without new safety concerns. The patients previously treated with placebo in the core study and who started atacicept 150 mg (PBO/atacicept group) showed an overall clinical improvement. However, the patients achieving the greatest proportion of SRI responses (SRI-4 58%, SRI-6 43.2%), low disease activity (47.7%), LLDAS (30.7%) and remission (26.1%) at 72 weeks were those taking 150 mg of atacicept continuously from the randomization in the core study. The percentages of patients in LDA and remission were significantly higher compared to those measured in the PBO/atacicept group.

### 5.6. Telitacicept

Telitacicept is a fusion protein consisting of a domain of TACI receptor and the crystallizable fragment (Fc) component of human IgG [106]. In China, telitacicept is approved in active SLE [107].

A phase IIb, randomized, placebo-controlled trial (Table 6) [108] studied telitacicept in combination with standard therapy compared to placebo in adults with SLE and a SELENA-SLEDAI score ≥8. Two hundred forty-nine patients were randomly assigned to four treatment groups. They received subcutaneous telitacicept 80 mg (n = 62), 160 mg (n = 63), 240 mg (n = 62), or placebo (n = 62) once a week as an add-on therapy. The primary efficacy endpoint was met. At week 48, in the groups treated with telitacicept, there were significantly greater proportions of SRI-4 responses than in the placebo group. The SRI-4 response was achieved by 71.0 % of the patients on telitacicept 80 mg (*p* < 0.0001), 68.3% of those on telitacicept 160 mg (*p* = 0.0001), 75.8% of those taking the highest dose (*p* < 0.0001). 33.9% of the patients in the placebo group were SRI-4 responders. Significantly higher proportions of telitacicept-treated patients experienced a reduction of ≥4 points in the SELENA-SLEDAI score compared to placebo-treated patients (75.8% with telitacicept 80 mg [p = 0.003], 77.8% with telitacicept 160 mg [*p* = 0.001], 79.0% with telitacicept 240 mg [*p* < 0.001], 50.0% with placebo). The frequency of AEs was similar between the groups.

A phase III trial (NCT04082416) [109] also based in China confirmed the efficacy and safety of telitacicept 160 mg. The study recruited adult SLE patients with a SELENA-SLEDAI score ≥8 who were given subcutaneous telitacicpet 160 mg once a week or placebo for 52 weeks. Three hundred thirty-five participants were randomized, of whom one hundred sixty-seven received telitacicept and one hundred sixty-eight received placebo. In the telitacicept group, 82.6% of patients achieved the SRI-4 response, compared to 38.1% of those treated with placebo (*p* <0.001). The higher response rate in telitacicept-treated patients was evident at week 4 and maintained throughout the study. Telitacicept was well tolerated, with only 7.2% of patients experiencing serious AEs versus 14.2% in the placebo group.

A phase III RCT (NCT05306574) of telitacicept is ongoing. Three hundred forty-one adolescent (≥12 years) and adult patients with moderate to severe SLE will be randomized to receive telitacicept 160 mg, telitacicept 240 mg, or placebo. The therapy will be administered once a week over the standard of care for 52 weeks. The primary endpoint will be the proportion of SRI-4 responders at 48 and 52 weeks.

An observational retrospective study of twenty Chinese patients [110], who were given telitacicept 160 mg weekly, provided early data on the efficacy and safety of telitacicept outside of the ideal setting of the RCTs. 80% of patients achieved the SRI-4 response, 90% succeeded in reducing the steroid dose by >25%, and none experienced severe flares. The therapy was well tolerated and only one patient discontinued the treatment because of AEs. An improvement in renal function was reported in patients with renal impairment, suggesting a potential role of telitacicept in lupus nephritis. Further investigations are needed.

### 5.7. Other Drugs

Tabalumab, an anti-BLyS monoclonal antibody, met its primary endpoint, achieving an SRI-5 response in one phase II trial (ILLUMINATE-2) [111] but not in a second trial (NCT01196091 [112]). Eli-Lilly decided not to take forward any further studies with this monoclonal. Blisibimod (anti-BLyS) and epratuzumab (anti-CD22) both gave encouraging phase II results [113,114] but did not meet their phase III endpoints [115,116] and no further trials with these monoclonals are planned.

## 6. Combination Therapies

The best studied combination therapy in SLE is rituximab and belimumab. Three phase II trials (Synbiose, CALIBRATE, and BEAT-LUPUS, Table 7) and the phase III BLISS-BELIEVE trial (Table 8) have explored the efficacy and safety of combining these two biologics.

The Synergic B-cell immunomodulation in SLE (Synbiose) trial [117] was a single-arm study investigating the immunological effects of sequential therapy with rituximab and belimumab. Sixteen patients with severe and refractory SLE were included. They were given intravenous rituximab 1 g at weeks 0 and 2, followed by intravenous belimumab at a dose of 10 mg/kg at weeks 4, 6, 8, and then monthly. The Synbiose trial demonstrated that belimumab, given after rituximab, inhibited the increasing levels of circulating BLyS detected in the serum of patients four weeks after the first infusion of rituximab. This effect was associated with a clinical improvement measured with disease activity indexes (SLEDAI and LLDAS). The median SLEDAI decreased significantly from 18 at baseline to 2 (*p* < 0.0001) at week 24 and thirteen patients achieved LLDAS at week 24.

A phase II, randomized, open-label (CALIBRATE) trial [118] assessed the safety of combining rituximab and belimumab in recurrent or refractory lupus nephritis. Forty-three adult patients, who previously had been treated with cyclophosphamide (CYC) or mycophenolate mofetil, were recruited and assigned to two groups. Every patient received CYC and intravenous rituximab at weeks 0 and 2. The patients of one group (RCB group) were given intravenous belimumab 10 mg/kg at weeks 4, 6, 8, and then monthly up to week 48. The patients in the other group (RC group) did not receive additional treatment beyond the CYC and rituximab. The study demonstrated that the combination of rituximab and belimumab was safe; the proportion of patients who experienced at least one severe AE did not differ significantly between the groups (23% in the RC group versus 9.5% in the RCB group, *p* > 0.05) at week 48. The combination of rituximab and belimumab was associated with a significantly lower B-cell count. This difference remained significant up to week 60 (*p* = 0.0012). Disappointingly, the RCB group did not have higher renal response rates compared to the RC group at all time points and the frequency of nonrenal flares was similar between the groups, suggesting that the addition of belimumab did not provide any clinical advantage. However, the trial was not powered to look for clinical change.

A 52 week, phase IIb, superiority RCT (BEAT-LUPUS) [119] studied the efficacy of belimumab given after rituximab infusions in patients with extrarenal and renal SLE. All fifty-two patients were given intravenous rituximab (1 g two weeks apart), after which they were randomly assigned to receive placebo or intravenous belimumab at the dose of 10 mg/kg at week 0, 2, 4, and then monthly up to week 52. The BEAT-LUPUS trial showed that, at 52 weeks, in the belimumab group there were significantly lower serum IgG anti-dsDNA antibody levels (primary endpoint of the study) than in the placebo group, 47 IU/mL versus 103 IU/mL of geometric mean, respectively (*p* < 0.001). The between-group difference in the serum anti-dsDNA levels was detectable since week 24 (*p* < 0.001). In contrast to the CALIBRATE trial, in the BEAT-LUPUS trial belimumab impacted significantly on the clinical outcome of the twenty-six belimumab-treated patients. Three had a severe flare compared to ten out of twenty-six in the placebo-treated group (*p* = 0.033). Considering safety, the frequency of adverse events was similar between the groups (two hundred forty-two AEs in the placebo group and two hundred forty-one AEs in the belimumab group). The BEAT-LUPUS trial thus demonstrated that the sequential therapy of belimumab given after rituximab is well tolerated and effective.

The BLISS-BELIEVE trial (NCT03312907) [120] was a 104-week, superiority, phase III, randomized controlled trial. It aimed to determine whether the combination of rituximab and belimumab was effective in achieving disease control and in reducing conventional therapy. Patients with severe kidney involvement or central nervous system lupus were excluded. Two hundred ninety-two patients were recruited and randomly assigned to three treatment arms. Patients in arm A (n = 72) received subcutaneous belimumab 200 mg weekly for 52 weeks and placebo infusions at weeks 4 and 6; in arm B (n = 144), instead of placebo infusions, patients received intravenous rituximab 1000 mg in addition to weekly subcutaneous belimumab injections. Those assigned to arm C (n = 76) were given subcutaneous belimumab 200 mg weekly plus standard of care up to 104 weeks. The primary endpoint was the proportion of patients who, at 52 weeks, achieved a state of disease control, defined as a SLEDAI-2K score ≤2, while keeping the steroid dose ≤5 mg/day and without immunosuppressants. The study did not meet its primary endpoint. There was no significant difference between the three arms in terms of achieving disease control. This was achieved by 16.7% of patients in arm A, 19.4% in arm B, and 25.5% in arm C.

A phase III open-label trial (Synbiose-2, NCT03747159) investigating the combination of belimumab and rituximab is ongoing. Seventy adults with severe extrarenal SLE and lupus nephritis will be enrolled and randomized to two treatment arms. One arm will receive subcutaneous belimumab at the dose of 200 mg weekly for two years, two intravenous infusion of rituximab 1000 mg at week 4 and 6, and the standard of care (steroids and mycophenolate mofetil). The other arm will receive only the standard of care. The primary endpoint of the study will be the proportion of treatment failures at 2 years. At 2 years the proportion of partial and complete renal responses will also be evaluated in patients with lupus nephritis. At frequent time points during the study the frequency of moderate to severe flares, the proportion of patients able to reduce concomitant immunosuppressants without flares, the disease activity captured by SLEDAI-2K, damage by using the System Lupus International Collaborating Clinics/American College of Rheumatology Damage Index for Systemic Lupus Erythematosus (SLICC), plus quality of life and safety will be assessed.

Apart from the sequential administration of rituximab and belimumab, elsubrutinib (a BTK inhibitor) and upadacitinib (a JAK inhibitor) have been investigated together as a combination therapy in a phase II trial (SLEek, NCT03978520), which has been recently completed. The SLEek study evaluated the proportion of SRI-4 responders who tapered the steroid dose to ≤10 mg of prednisone equivalent at week 24 and, as secondary endpoints, the achievement of SRI-4, BICLA, LLDAS, the number of flares, and the change in steroids dose. The results are awaited.

## 7. Pediatric SLE

The treatment of children with SLE is still challenging due to the small representation of this age group in clinical trials. Indeed, RCT eligibility criteria often require patients to be adults. As mentioned above, in pediatric lupus patients with renal and extra-renal involvement only intravenous belimumab has been approved by the FDA as a biological therapy. A phase II trial (PLUTO, NCT01649765) [73] showed that pediatric lupus patients (5–17 years) treated with intravenous belimumab had more favorable clinical outcomes compared to those treated with placebo, with 52.8% SRI-4 responders at 52 weeks versus 43.6%. The p-value was not reported, since the study did not enroll an adequate number of patients to detect a statistically significant difference. The subcutaneous formulation of belimumab has not yet been approved in children and adolescents. A phase II trial (NCT04179032) evaluating the pharmacokinetics and pharmacodynamics of subcutaneous belimumab in pediatric patients aged 5–17 years is currently ongoing. Among the phase II trials reported in this review, the only one that enrolled adolescents was the APRIL-SLE trial. However, a greater number of phase III trials will include adolescent patients, notably the studies evaluating dapirolizumab (PHOENYCS GO, NCT04294667, and NCT04976322: ≥16 years), telitacicept (NCT05306574, 12–70 years), and the OBILUP trial of obinutuzumab (NCT04702256), which will enroll patients aged at least 14 years. These trials will provide useful data on the efficacy of emerging drugs in adolescents. Given that none of these studies will enroll patients aged less than twelve years, the data on the efficacy in children will continue to be lacking.

## 8. Resume of Drugs in Phase I Clinical Trials

Several ongoing phase I clinical trials are evaluating the safety profile, pharmacokinetics, and pharmacodynamics of new potential drugs. We will briefly summarize the biological drugs that are in phase I trials: mosunetuzumab, itolizumab, CM313, and DS-7011a.

Mosunetuzumab, a CD20/CD3 bispecific antibody, engages T cells and redirects them to attack CD20+ B-cells [121]. It has been recently approved in Europe for the treatment of follicular lymphoma [122]. A phase I trial (NCT05155345) will evaluate the tolerance and the effect on the B-cell count of two different doses of mosunetuzumab in patients with active SLE (SLEDAI-2K ≥ 4).

Another biological drug targeting a molecule expressed on T cells is itolizumab. This is a humanized IgG1 monoclonal antibody which binds CD6 and downregulates T cell activation [123]. A phase I trial (EQUALISE, NCT04128579) will explore the safety profile of different doses of itolizumab in two patient cohorts, one with non-renal lupus and the other focusing on patients with proliferative lupus nephritis (class III or IV).

CM313 is a monoclonal antibody directed against CD38, a glycoprotein expressed mainly on plasma cells [124]. A phase Ib/II, randomized, double-blind, placebo-controlled trial (NCT05465707) will evaluate its safety, pharmacokinetics, and preliminary efficacy in patients with SLE who will receive different doses of CM313 (2 mg/kg, 4 mg/kg, 8 mg/kg, 16 mg/kg).

DS-7011a is a monoclonal antibody targeting the Toll-like receptor 7 (TLR7), which has been implicated in the promotion of autoimmune responses [125]. A phase Ib/II, randomized, double-blind, placebo-controlled trial (NCT05638802) will enroll twenty-four patients, to be randomized to receive placebo or intravenous infusions of DS-7011a at a dose of 20 mg/kg every 4 weeks. The proportion of AEs, multiple pharmacokinetic parameters, the change in disease activity, in the autoantibodies, and in complement component levels will be measured.

## 9. Discussion

In this review, the trials of biological therapies approved or under evaluation in lupus patients have been summarized. Many factors are thought to have limited the development of biologics in SLE, so that the treatment remains mostly based on conventional immunosuppressants, which have several adverse events and can increase the risk of damage accrual, especially in the case of corticosteroids.

Belimumab and anifrolumab are the two biologics approved by the FDA in lupus patients. However, few patients can benefit from these drugs, because of the limitations in their prescription. Rituximab has never been approved by regulatory agencies (except for NHS England in the UK), but it is recommended as an off-label treatment in case of severe or refractory SLE. B-cells have long represented an attractive target in SLE since they are thought to play a central role in the pathogenesis. Combining B-cell depletion with rituximab and anti-BLyS treatment with belimumab has proved effective in reducing the flare rate in the BEAT-LUPUS trial [119], without concerns regarding the safety. This supported the hypothesis that the EXPLORER [58] and LUNAR [59] trials failed because of the surge of BLyS observed after the infusions of rituximab [61]. Unfortunately, the BLISS-BELIEVE study was not successful so that the future for this form of combination therapy remains uncertain.

More encouragingly, six new biological drugs showed promising results in phase II trials and will be soon evaluated in phase III trials. The majority of the phase II trials included patients without lupus nephritis. Dapirolizumab, deucravacitinib, litifilimab, and telitacicept improved multiple clinical outcome measures in patients with extra-renal SLE and their safety profile was considered acceptable. An increase frequency of cutaneous AEs was noted during treatment with deucravacitinib, but these were not considered severe.

With regard to lupus nephritis, obinutuzumab and atacicept have been evaluated in phase II trials. In the NOBILITY trial [88], a rapid and sustained B-cell depletion and a higher proportion of renal responses were reported with obinutuzumab compared to placebo. If phase III trials confirm the beneficial effect reported, obinutuzumab could potentially replace rituximab in the treatment of refractory lupus nephritis. Indeed, fully humanized monoclonal antibodies compared to chimeric monoclonal antibodies have a lower risk of allergic reactions, a common issue that has often limited the use of rituximab. However, it must be acknowledged that the NOBILITY trial had some limitations, notably the limited sample size of one hundred twenty-five participants and the prespecified alpha level of 0.2.

The efficacy of atacicept in LN could not be evaluated in the phase II trial since it was discontinued prematurely due to the inadvertent concern about the infectious risk [103]. Two deaths due to infection occurred in patients on atacicept 150 mg in the APRIL-SLE trial with a subsequent termination of this treatment arm [101]. To note, the number of infections was not greater than the one observed in other previous lupus trials that were not discontinued and achieved significant responses. Furthermore, a subsequent integrated analysis of atacicept trials has provided reassurance that this monoclonal is not linked to an increased infection risk [126]. Despite the failure of the phase II trials in extra-renal SLE, it seemed that atacicept might be effective in a subgroup of patients with active serology and high disease activity.

## 10. Conclusions

The development of biologics in SLE clearly lags behind the therapeutic successes seen in other rheumatological diseases, with only two biologics approved in lupus patients. The analysis of the trials of rituximab, belimumab, and anifrolumab has taught the investigators that an accurate design of the studies and a careful choice of the outcome measures are vital to detect the potential benefit of new biological drugs in SLE. In addition, in such a heterogeneous disease as SLE, the beneficial effects of some drugs might not be evident in the whole population of lupus patients, but only in specific subgroups. The experience gained from the lupus trials should now allow better design of phase II and III studies which should eventually allow more effective biological drugs to emerge and take their place in the pantheon of lupus therapy.

## 11. Future directions

New effective biological drugs could at least partially replace conventional immunosuppressants, potentially leading to a reduction in organ damage and a subsequent improvement in the prognosis and patients’ quality of life. Moreover, with the expansion of the therapeutic armamentarium, different approaches to the disease would be feasible, notably combination and sequential therapies. Biological drugs targeting different molecules could also be the basis of a personalized medicine, tailored to the individual patient and their immunological alterations.

## Figures and Tables

**Table 1:** Phase II trial of obinutuzumab.

Obinutuzumab	
Clinical trial name	NOBILITY (NCT02550652)
Primary endpoint What was it? Was it achieved?	CRR at 52 W CRR, n (%) OBI vs. PBO: 22 (35) vs. 14 (23), *p* = 0.115 (statistically significant, alpha level 0.2)
Eligibility criteria	-SLE (ACR classification criteria—1997) -Class III or IV LN -UPCR >1 -eGFR ≥30 mL/min/1.73 m^2^ -18–75 years
Treatment scheme	IV OBI 1000 mg or IV PBO on day 1, W2, W24, W26 + MMF + GC
Number of patients who received the treatment	63 (OBI) vs. 62 (PBO)
Study duration	104 weeks
Key secondary endpoints	Clinical -ORR at 52 W, n (%) OBI vs. PBO: 35 (56) vs. 22 (36), *p* = 0.025 -ORR at 104 W, n (%) OBI vs. PBO: 34 (54) vs. 18 (29), *p* = 0.005 -CRR at 104 W in patients with class IV LN, n (%) OBI vs. PBO: 23 (47) vs. 7 (16), *p* = 0.001 -CRR at 104 W in patients with BL UPCR ≥3, n (%) OBI vs. PBO: 8 (31) vs. 2 (10), *p* = 0.098 Laboratory -Change in C3 from BL at 52 W, mean (SE) OBI vs. PBO: 30 (3.4) vs. 12 (3.5), *p* < 0.001 -Change in C3 from BL at 104 W, mean (SE) OBI vs. PBO: 29 (3.4) vs. 11 (3.4), *p* < 0.001 -Change in C4 from BL at 52 W, mean (SE) OBI vs. PBO: 9.7 (1.3) vs. 0.8 (1.3), *p* < 0.001 -Change in C4 from BL at 104 W, mean (SE) OBI vs. PBO: 9.6 (1.3) vs. 0.4 (1.3), *p* < 0.001 -Change in log anti-dsDNA titre from BL at 52 W, mean (SE) OBI vs. PBO: −0.91 (0.12) vs. −0.10 (0.12), *p* < 0.001 -Change in log anti-dsDNA titre from BL at 104 W, mean (SE) OBI vs. PBO: −1.1 (0.13) vs. −0.05 (0.13), *p* < 0.001
Safety	Any AE, n (%) OBI vs. PBO: 58 (91) vs. 54 (89) Serious infectious AE, n (%) OBI vs. PBO: 5 (8) vs. 11 (18) Infusion-related reactions, n (%) OBI vs. PBO: 10 (16) vs. 6 (10)

ACR: American College of Rheumatology. AE: adverse events. BL: baseline. CRR: complete renal response. eGFR: estimated glomerular filtration rate. GC: glucocorticoids. IV: intravenous. LN: lupus nephritis. MMF: mycophenolate mofetil. OBI: obinutuzumab. ORR: overall renal response. PBO: placebo. SE: standard error. SLE: systemic lupus erythematosus. UPCR: urine protein to creatinine ratio. W: week.

**Table 2:** Phase II trial of dapirolizumab.

Dapirolizumab	
Clinical trial name and/or identification	RISE (NCT02804763)
Primary endpoint What was it? Was it achieved?	Identifying a dose-response relationship. Responses defined by BICLA at W24. Not met (*p* = 0.07)
Eligibility criteria	-SLE (SLICC classification criteria) -Positive anti-dsDNA antibodies and/or low complement and/or an ANA titre 1:80, in combination with historical positivity for anti-dsDNA and/or positivity for anti-ENA [anti-Sm, anti-SSA, anti-SSB or anti-RNP] -Moderate to severe disease activity: ≥1 BILAG A or ≥2 BILAG B domains; SLEDAI-2K ≥ 6, and a SLEDAI-2K ≥4 excluding points from laboratory values 4 -≥18 years
Key exclusion criteria	-class III or IV LN -severe neuropsychiatric SLE -history of thromboembolic events within 12 months of screening
Treatment scheme	DZP IV or PBO IV every 4 weeks up to W20
Number of patients who received the treatment	45 PBO, 45 DZP 6 mg/kg, 45 DZP 24 mg/kg, 47 DZP 45 mg/kg
Study duration	48 W (24 W treatment period + 24 W observational period)
Key secondary endpoints	Clinical * -BILAG 2004 W24 (mean change from BL PBO, DZP 6 mg/kg, DZP 24 mg/kg, DZP 45 mg/kg): −10.5, −10.9, −14.0, −12.0 (*p* <0.05) -BILAG 2004 W48 (mean change from BL PBO, DZP 6 mg/kg, DZP 24 mg/kg, DZP 45 mg/kg): −10.6, −11.5, −12.9, −12.7 (*p* <0.05)
Safety	Any TEAE, n (%) (PBO, DZP 6 mg/kg, DZP 24 mg/kg, DZP 45 mg/kg): 28 (62.2), 29 (64.4), 35 (77.8), 34 (72.3) Serious TEAEs, n (%) (PBO, DZP 6 mg/kg, DZP 24 mg/kg, DZP 45 mg/kg): 5 (11.1), 2 (4.4), 4 (8.9), 5 (10.6) Thromboembolic events, n (%) (PBO, DZP 6 mg/kg, DZP 24 mg/kg, DZP 45 mg/kg): 3 (6.7), 0, 1 (2.2), 0

* only statistically significant clinical secondary endpoints have been reported. Ab: antibodies. BICLA: BILAG-based Composite Lupus Assessment. BILAG: British Isles Lupus Assessment Group index. BL: baseline. CLASI-A: Cutaneous Lupus Erythematosus Disease Area and Severity Index–Activity. DZP: dapirolizumab. IV: intravenous. LN: lupus nephritis. PGA: Physician’s Global Assessment. PBO: placebo. SE: standard error. SLE: systemic lupus erythematosus. SLEDAI-2K: Systemic Lupus Erythematosus Disease Activity Index-2000. SLICC: System Lupus International Collaborating Clinics/American College of Rheumatology Damage Index for Systemic Lupus Erythematosus. SRI: Systemic lupus erythematosus Response Index. TEAE: treatment-emergent adverse event. W: week.

**Table 3:** Phase II trial of deucravacitinib.

Deucravacitinib	
Clinical trial name and/or identification	PAISLEY (NCT03252587)
Primary endpoint What was it? Was it achieved?	SRI-4 response at W32 SRI-4 responses, n (%), PBO vs. DEU 3 mg BID: 31 (34.4) vs. 53 (58.2), *p* <0.001 SRI-4 responses, n (%), PBO vs. DEU 6 mg BID: 31 (34.4) vs. 46 (49.5), *p* = 0.02 SRI-4 responses, n (%), PBO vs. DEU 12 mg QD: 31 (34.4) vs. 40 (44.9), *p* = 0.08
Eligibility criteria	-SLE (SLICC classification criteria) -≥1 positive test for ANA, anti-dsDNA Ab, anti-Sm -≥1 BILAG A or ≥2 BILAG B from the musculoskeletal or mucocutaneous domain -SLEDAI-2K ≥6 -18–75 years -taking ≥1 antimalarial or immunosuppressant
Key exclusion criteria	-active, severe LN -active neuropsychiatric SLE -history of herpes zoster, herpes simplex, or influenza infection within 12 weeks before randomization -history of disseminated or complicated herpes zoster infection
Treatment scheme	4 arms -PBO + SOC -DEU 3 mg BID for 48 W + SOC -DEU 6 mg BID for 48 W + SOC -DEU 12 mg QD for 48 W + SOC
Number of patients who received the treatment	90 PBO, 91 DEU 3 mg BID, 93 DEU 6 mg BID, 89 DEU 12 mg QD
Study duration	48 W
Key secondary endpoints	Clinical -SRI-4 responders 48 W, n (%) PBO vs. DEU 3 mg BID: 31 (34.4) vs. 52 (57.1), *p* <0.001 -BICLA responders 48 W, n (%) PBO vs. DEU 3 mg BID: 23 (25.6) vs. 43 (47.3), *p* = 0.001 -LLDAS responders 48 W, n (%) PBO vs. DEU 3 mg BID: 12 (13.3) vs. 33 (36.3), *p* <0.001 -CLASI-50 responders 48 W, n (%) PBO vs. DEU 3 mg BID: 4 (16.7) vs. 16 (69.6), *p* <0.001 -Mean change from BL in the joint count, n (%) PBO vs. DEU 3 mg BID: −7.6 vs. −8.9, *p* = 0.001
Safety	Any AE, n (%) PBO, DEU 3 mg BID, DEU 6 mg BID, DEU 12 mg QD: 79 (87.8), 85 (93.4), 81 (87.1), 75 (84.3) SAEs, n (%) PBO, DEU 3 mg BID, DEU 6 mg BID, DEU 12 mg QD: 11 (12.2), 7 (7.7), 8 (8.6), 7 (7.9) Herpes zoster, n (%) PBO, DEU 3 mg BID, DEU 6 mg BID, DEU 12 mg QD: 4 (4.4), 3 (3.3), 3 (3.2), 2 (2.2) Cutaneous AEs, % PBO, DEU 3 mg BID, DEU 6 mg BID, DEU 12 mg QD: 13.3, 16.5, 34.4, 33.7

Ab: antibodies. AE: adverse event. ANA: antinuclear antibodies. BICLA: BILAG-based Composite Lupus Assessment. BID: twice a day. BILAG: British Isles Lupus Assessment Group index. BL: baseline. CLASI: Cutaneous Lupus Erythematosus Disease Area and Severity Index. DEU: deucravacitinib. IV: intravenous. LLDAS: Lupus Low Disease Activity State. LN: lupus nephritis. PGA: Physician’s Global Assessment. PBO: placebo. QD: each day. SAEs: serious adverse events. SLE: systemic lupus erythematosus. SLEDAI-2K: Systemic Lupus Erythematosus Disease Activity Index-2000. SLICC: System Lupus International Collaborating Clinics/American College of Rheumatology Damage Index for Systemic Lupus Erythematosus. SOC: standard of care. SRI: Systemic lupus erythematosus Response Index. W: week.

**Table 4:** Phase II trial of litifilimab.

Litifilimab	
Clinical trial name and/or identification	LILIAC (NCT02847598)
Primary endpoint What was it? Was it achieved?	Control of joint involvement at W24 LSM (±SE) absolute changes from BL in the total number of active joints litifilimab vs. PBO: −15.0 ± 1.2 vs. −11.6 ± 1.3. LSM difference litifilimab vs. PBO: −3.4 (*p* = 0.04)
Eligibility criteria	-SLE (ACR classification criteria 1997) -ANA ≥ 1:80 and/or anti-dsDNA Ab ≥ 30 UI/mL -CLASI-A ≥ 8 (protocol version 1) -≥1 active skin lesion (protocol version 2, no requirements for CLASI-A) -≥4 tender and swollen joints (protocol version 2) -SLEDAI-2K ≥ 4 -18–75 years
Key exclusion criteria	-active LN -active neuropsychiatric SLE
Treatment scheme	PBO sc or litifilimab 450 mg sc at W 0, 2, 4, 8, 12, 16, 20 + SOC
Number of patients who received the treatment	56 PBO, 64 litifilimab 450 mg
Study duration	24 W treatment period + 12W observation period
Key secondary endpoints	Clinical -Decrease of ≥7 points in CLASI-A at W24, LSM difference (95% CI) litifilimab vs. PBO: 21.6 (0.1 to 43.1) -SRI-4 responders at W24, LSM difference (95% CI) litifilimab vs. PBO: 26.4 (9.5 to 43.2) -Change in SLEDAI-2K at W24, LSM difference (95% CI) litifilimab vs. PBO: –1.7 (–3.0 to –0.5)
Safety	Any AE, n (%) pooled litifilimab vs. PBO: 45 (59) vs. 38 (68) Serious AEs, n (%) pooled litifilimab vs. PBO: 4 (5) vs. 6 (11)

Ab: antibodies. ACR: American College of Rheumatology. AE: adverse event. ANA: antinuclear antibodies. BL: baseline. CLASI-A: Cutaneous Lupus Erythematosus Disease Area and Severity Index—Activity. LLDAS: Lupus Low Disease Activity State. LN: lupus nephritis. LSM: least-squares mean. PGA: Physician’s Global Assessment. PBO: placebo. QD: each day. SAEs: serious adverse events. Sc: subcutaneous. SE: standard error. SLE: systemic lupus erythematosus. SLEDAI-2K: Systemic Lupus Erythematosus Disease Activity Index-2000. SOC: standard of care. SRI: Systemic lupus erythematosus Response Index. W: week.

**Table 5:** Phase II trials of atacicept.

Atacicept		
Clinical trial name and/or identification	APRIL-SLE (NCT00624338)	ADDRESS II (NCT01972568)
Primary endpoint What was it? Was it achieved?	The proportion of BILAG A or B flares Not met. Flare rate atacicept 75 mg vs. PBO: 58% vs. 54% (*p* = 0.543) PBO vs. atacicept 150 mg (post-hoc analysis): Flare rate: 37% vs. 54% (*p* = 0.002)	SRI-4 responders at 24 W Not met. Atacicept 75 mg vs. PBO: 57.8% vs. 44.0% (*p* = 0.045) Atacicept 150 mg vs. PBO: 53.8% vs. 44.0% (*p* =0.121) Using day 1 as BL (sensitivity analysis): *p* < 0.05 for SRI-4 of both atacicept doses vs. PBO Serologically active patients: -SRI-4 responses atacicept 75 mg vs. PBO: 62.1% vs. 24.1% (*p* = 0.003) -SRI-4 responses atacicept 150 mg vs. PBO: 61.5% VS. 24.1% (*p* = 0.002) HDA at BL: SRI-4 responses atacicept 150 mg vs. PBO: 62.7% vs. 42.3% (*p* = 0.029)
Eligibility criteria	-≥4/11 ACR classification criteria (1997) -ANA ≥1:80 and/or anti-dsDNA Ab ≥30 IU/mL -active SLE (BILAG A or B that led to a change in steroid dose) -SLEDAI-2K ≥ 6 -≥16 years	-≥4/11 ACR classification criteria (1997) -ANA ≥1:80 and/or anti-dsDNA Ab ≥30 IU/mL -≥1 BILAG A or ≥2 BILAG B from the -SLEDAI-2K ≥ 6 -disease duration ≥ 6 months -≥18 years
Key exclusion criteria	-therapy with CYC, MMF, CNIs, LEF, 6-MP, or thalidomide within 3 months of the screening -severe CNS lupus -active moderate-to-severe GN	-CYC within 3 months of the screening -severe CNS lupus -active severe GN
Treatment scheme	3 arms: -PBO + SOC -Atacicept 75 mg sc BIW for 4 weeks, then QW + SOC -Atacicept 150 mg sc BIW for 4 weeks, then QW + SOC (the enrollment in this group was terminated prematurely)	3 arms: -PBO + SOC -Atacicept 75 mg sc QW + SOC -Atacicept 150 mg sc QW + SOC
Number of patients who received the treatment	157 PBO, 159 atacicept 75 mg, 145 atacicept 150 mg	100 PBO, 102 atacicept 75 mg, 104 atacicept 150 mg
Study duration	52 W	24 W treatment period + 24 W safety follow-up period
Key secondary endpoints	Clinical -Time to first flare atacicept 75 mg vs. PBO (ITT analysis set), HR (95% CI): 0.98 (0.69 to 1.40), *p* = 0.929 -Time to first flare atacicept 75 mg vs. PBO (PC analysis set), HR (95% CI): 0.83 (0.53 to 1.29), *p* = 0.404 PBO vs. atacicept 150 mg (post-hoc analysis): -Time to first flare atacicept 150 mg vs. PBO (ITT analysis set), HR (95% CI): 0.56 (0.36 to 0.87), *p* = 0.009 -Time to first flare atacicept 150 mg vs. PBO (PC analysis set), HR (95% CI): 0.41 (0.24 to 0.70), *p* = 0.001 Immunological -Anti-dsDNA Ab change from BL, atacicept 150 mg, atacicept 75 mg, PBO: −38%, −31%, +14% -C3 change, LSM change atacicept 75 mg vs. PBO: 0.076 (*p* < 0.001) -C3 change, LSM change atacicept 150 mg vs. PBO: 0.138 (*p* < 0.001) -C4 change, LSM change atacicept 75 mg vs. PBO: 0.046 (*p* < 0.001) -C4 change, LSM change atacicept 150 mg vs. PBO: 0.066 (*p* < 0.001)	SRI-6 responders atacicept 150 mg vs. PBO: 54.9% vs. 28.8% (*p* = 0.005) -BILAG-A flares atacicept 75 mg vs. PBO (all pts): HR 0.24 (95% CI 0.07 to 0.87, *p* = 0.019) -BILAG-A flares atacicept 150 mg vs. PBO (all pts): HR 0.54 (95% CI 0.21 to 1.39, *p* = 0.198) -BILAG-A flares atacicept 75 mg vs. PBO (HDA): HR 0.08 (95% CI 0.01 to 0.59, *p* = 0.002) -BILAG-A flares atacicept 150 mg vs. PBO (HDA): HR 0.32 (95% CI 0.10 to 0.99, *p* = 0.038) Immunological: -Anti-dsDNA Ab median % change from BL atacicept 75 mg, 150 mg, PBO: −23.6%, −28.2%, +16.0% -C3 median % change from BL atacicept 75 mg, 150 mg, PBO: +5.3%, +22.1%, +1.5% -C4 median % change from BL atacicept 75 mg, 150 mg, PBO: +64.5%, +128.6%, 0
Safety	Any AE (PBO, atacicept 75 mg, atacicept 150 mg): 79.9%, 83.4%, 83.3% Serious AEs (PBO, atacicept 75 mg, atacicept 150 mg): 17.5%, 19.1%, 16.0%	TEAEs (PBO, atacicept 75 mg, atacicept 150 mg): 72.0%, 81.4%, 80.8% Serious TEAEs (PBO, atacicept 75 mg, atacicept 150 mg): 12.0%, 5.8%, 8.8%

Ab: antibodies. ACR: American College of Rheumatology. AE: adverse event. ANA: antinuclear antibodies. BILAG: British Isles Lupus Assessment Group index. BIW: twice a week. BL: baseline. CI: confidence interval. CLASI: Cutaneous Lupus Erythematosus Disease Area and Severity Index. CNIs: calcineurin inhibitors. CNS: central nervous system. CYC: cyclophosphamide. GN: glomerulonephritis. HDA: high disease activity. HR: hazard ratio. ITT: intention-to-treat. LEF: leflunomide. LSM: least square mean. MMF: mycophenolate mofetil. MP: mercaptopurine. PBO: placebo. PC: potential completer. PGA: Physician’s Global Assessment. Pts: patients. QD: each day. QW: weekly. Sc: subcutaneous. SLE: systemic lupus erythematosus. SLEDAI-2K: Systemic Lupus Erythematosus Disease Activity Index-2000. SLICC: System Lupus International Collaborating Clinics/American College of Rheumatology Damage Index for Systemic Lupus Erythematosus. SOC: standard of care. SRI: Systemic lupus erythematosus Response Index. TEAE: treatment-emergent adverse event. W: week.

**Table 6:** Phase II trial of telitacicept.

Telitacicept	
Clinical trial name and/or identification	NCT02885610
Primary endpoint What was it? Was it achieved?	SRI-4 responders at 48 W -SRI-4 responders (%) telitacicept 80 mg vs. PBO: 71.0 vs. 33.9 (*p* < 0.0001) -SRI-4 responders (%) telitacicept 160 mg vs. PBO: 68.3 vs. 33.9 (*p* = 0.0001) -SRI-4 responders (%) telitacicept 240 mg vs. PBO: 75.8 vs. 33.9 (*p* <0.0001)
Eligibility criteria	-≥4/11 ACR classification criteria (1997) -SELENA-SLEDAI ≥ 8 -positive ANA and/or anti-dsDNA Ab -18–65 years
Key exclusion criteria	-severe LN -CNS involvement
Treatment scheme	4 arms: -PBO + SOC -telitacicept 80 mg sc QW for 48 weeks + SOC -telitacicept 160 mg sc QW for 48 weeks + SOC -telitacicept 240 mg sc QW for 48 weeks + SOC
Number of patients who received the treatment	62 telitacicept 80 mg, 63 telitacicept 160 mg, 62 telitacicept 240 mg, 62 PBO
Study duration	48 W
Key secondary endpoints	Proportion of patients with ≥ 4 points reduction in SELENA-SLEDAI W48: -telitacicept 80 mg vs. PBO (%): 75.8 vs. 50.0 (*p* = 0.003) -telitacicept 160 mg vs. PBO (%): 77.8 vs. 50.0 (*p* = 0.001) -telitacicept 240 mg vs. PBO (%): 79.0 vs. 50.0 (*p* <0.001) Proportion of patients without worsening PGA W48: -telitacicept 80 mg vs. PBO (%): 96.8 vs. 75.8 (*p* <0.001) -telitacicept 160 mg vs. PBO (%): 92.1 vs. 75.8 (*p* = 0.013) -telitacicept 160 mg vs. PBO (%): 96.8 vs. 75.8 (*p* <0.001)
Safety	Any AE (%): 82.3 (PBO), 90.3 (telitacicept 80 mg), 92.1 (telitacicept 160 mg), 93.5 (telitacicept 240 mg) (*p* >0.05) SAEs (%): 16.1 (PBO), 12.9 (telitacicept 80 mg), 15.9 (telitacicept 160 mg), 12.9 (telitacicept 240 mg) (*p* >0.05)

Ab: antibodies. ACR: American College of Rheumatology. AE: adverse event. ANA: antinuclear antibodies. CNS: central nervous system. LN: lupus nephritis. PGA: Physician’s Global Assessment. PBO: placebo. QW: weekly. SAEs: serious adverse events. SELENA-SLEDAI: Safety of Estrogen in Lupus National Assessment—Systemic Lupus Erythematosus Disease Activity Index. SOC: standard of care. SRI: Systemic lupus erythematosus Response Index. W: week.

**Table 7:** Phase II trials of rituximab and belimumab (combination therapy).

Rituximab + Belimumab			
Clinical trial name and/or identification	CALIBRATE (NCT02260934)	BEAT-LUPUS	Synbiose (NCT02284984)
Primary endpoint What was it? Was it achieved?	Safety: % of pts with AE (≥ grade 3) up to W48 23% RC vs. 9.5% RCB (*p* >0.05)	Serum IgG anti-dsDNA levels at W52 Anti-dsDNA levels, geometric mean (IU/mL) [95% CI], BEL vs. PBO: 47 [25–88] vs. 103 [49–213], *p* < 0.001	Immunological effects of RTX + BEL at W24 -BlyS decreased to 0.15 ng/mL [0.05–0.4], *p* < 0.01 -anti-dsDNA Ab decreased to 57 IU/mL [10–374], *p* = 0.0004 -NETs formation reduced to 1.9-fold increase compared to controls [0.4–6.1] (*p* = 0.0006)
Eligibility criteria	-SLE ACR or SLICC criteria -ANA and/or anti-dsDNA Ab + -≥18 years -recurrent or refractory LN -prior therapy with CYC or MMF	-SLE -18–75 years -anti-dsDNA Ab + at least once in the 5 years before the screening -failure of conventional therapy	-SLE (ACR criteria 1997) -≥18 years -severe SLE flare or refractory disease -ANA ≥1:80, anti-dsDNA Ab ≥30 IU/mL, hypocomplementemia
Key exclusion criteria	-prior therapy with RTX -prior therapy with another B-cell biologic therapy within the prior 12 months	-BILAG A flare in CNS -prior therapy with biological drugs (except RTX) -low IgG or IgA levels	-significant B-cell depletion -significant hypogammaglobulinemia
Treatment scheme	All pts: MP 100 mg + RTX 1000 mg + CYC IV 750 mg W0, W2 At W4 randomization: -RTX + CYC followed by BEL 10 mg/kg IV W4, 6, 8, and then every 4 weeks (RCB group) -RTX + CYC	All pts: RTX IV 1000 mg two weeks apart 4–8 W after RTX randomization: -BEL 10 mg/kg IV W0, 2, 4, and then every 4 weeks up to W52 -PBO	Single arm: RTX 1000 mg IV W0 and W2 + BEL 10 mg/kg W4, 6, 8, then monthly.
Number of patients who received the treatment	21 RCB group, 22 RC group	26 RTX + BEL, 26 RTX + PBO	16
Study duration	96 W	52 W	24 W
Key secondary endpoints	-ORR W48, n (%) RCB vs. RC: 11 (52) vs. 9 (41), *p* = 0.452 -n of B-cells, geometric mean (cells/µL) [95% CI] W60, RCB vs. RC: 11 [6–20] vs. 53 [26–109], *p* = 0.0012 -pts with B-cell reconstitution W24, n RCB vs. RC: 0 vs. 5, *p* = 0.041 -median IgG (mg/dl), RCB vs. RC: 904.5 vs. 1410.0, *p* = 0.022	-severe flares W52, n, BEL vs. PBO: 3 vs. 10, HR: 0.27 (95% CI, 0.07–0.98), unadjusted log-rank *p* = 0.003 -B-cell count W52, geometric mean [95% CI], BEL vs. PBO: 0.012 [0.006–0.014] vs. 0.037 [0.021–0.081], *p* = 0.031	-median SLEDAI BL vs. W24: 18 vs. 2, *p* < 0.0001 -median GC dose (mg/day) BL vs. W24: 60 [5–60] vs. 7.5 [5–12.5], *p* = 0.001
Safety	TEAEs ≥ grade 2, n (%) [95% CI], RCB vs. RC: 21 (100) [0.00–16.11] vs. 22 (100) [0.00–15.44] Serious TEAEs, n (%) [95% CI], RCB vs. RC: 4 (19) [5.45–41.91] vs. 11 (50) [28.22–71.78] Infectious TEAEs ≥grade 3, n (%) [95% CI], RCB vs. RC: 2 (10) [1.17–30.38] vs. 6 (27) [10.73–50.22]	All AEs, n of pts (%), BEL vs. PBO: 24 (92) vs. 24 (92) All AEs, n of events (%), BEL vs. PBO: 241 vs. 242 SAEs, n (%), BEL vs. PBO: 6 (23) vs. 6 (23)	Any AE, n = 41 Major infections, n (%) 1 (2) Minor infections, n (%) 15 (37%)

Ab: antibodies. ACR: American College of Rheumatology. AE: adverse event. ANA: antinuclear antibodies. BEL: belimumab. BILAG: British Isles Lupus Assessment Group index. BIW: twice a week. BL: baseline. BLys: B lymphocyte stimulator. CI: confidence interval. CNS: central nervous system. CYC: cyclophosphamide. HR: hazard ratio. IV: intravenous. MP: mehylprednisolone. NETs: neutrophil extracellular traps. ORR: overall renal responses (complete renal responses + partial renal responses). PBO: placebo. Pts: patients. RC: rituximab + cyclophosphamide group. RCB: rituximab + cyclophosphamide + belimumab group. RTX: rituximab. SLE: systemic lupus erythematosus. SLEDAI: Systemic Lupus Erythematosus Disease Activity Index. SLICC: System Lupus International Collaborating Clinics/American College of Rheumatology Damage Index for Systemic Lupus Erythematosus. SAEs: serious adverse events. SRI: Systemic lupus erythematosus Response Index. TEAE: treatment-emergent adverse event. W: week.

**Table 8:** Phase III trial of rituximab and belimumab (combination therapy).

Rituximab + belimumab	
Clinical trial name and/or identification	BLISS-BELIEVE (NCT03312907)
Primary endpoint What was it? Was it achieved?	Disease control (SLEDAI-2K = 2) at W52 Not met. Proportion of pts with disease control, n (%) BEL/PBO, BEL/RTX, BEL/ST: 12 (16.7), 28 (19.4), 12 (25.5) -Disease control BEL/RTX vs BEL/PBO, observed difference [OR (95% CI)]: 2.78 [1.27 (0.60-2.71)], p = 0.5342 -Exploratory comparison: BEL/RTX vs BEL/ST, observed difference [OR (95% CI)]: -6.09 [0.71 (0.32–1.54)]
Eligibility criteria	-SLE (ACR criteria) -SLEDAI-2K ≥6 -ANA and/or anti-dsDNA Ab + -≥18 years
Key exclusion criteria	-severe lupus kidney disease -severe active CNS lupus -evidence of serious suicide risk -IgA <10 mg/dl -IgG <250 mg/dl
Treatment scheme	3 arms: -BEL 200 mg sc QW for 52 weeks + PBO IV W4 and W6 (BEL/PBO) -BEL 200 mg sc QW for 52 weeks + RTX 1000 mg IV at W4 and W6 (BEL/RTX) -BEL 200 mg sc QW + ST for 104 weeks (BEL/ST).
Number of patients who received the treatment	72 BEL/PBO, 144 BEL/RTX, 76 BEL/ST
Study duration	104 W
Key secondary endpoints	-pts with clinical remission at W64, observed difference [OR (95% CI)] BEL/RTX vs BEL/PBO: 0.69 [1.12 (0.33–3.78)], *p* = 0.8582 BEL/RTX vs BEL/ST: −4.39 [0.53 (0.17–1.70)] -disease control at W104, observed difference [OR (95% CI)] BEL/RTX vs BEL/PBO: 4.17 [1.64 (0.57–4.72)], *p* = 0.3613 BEL/RTX vs BEL/ST:−10.17 [0.45 (0.19–1.09)] -disease control duration by W52, treatment difference (95% CI) BEL/RTX vs BEL/PBO: 47.0 (8.0–86.0), *p* = 0.0188 BEL/RTX vs BEL/ST: 18.2 (−22.3–58.7) -decrease in anti-dsDNA Ab from BL at W52, BEL/RTX vs BEL/PBO: *p* < 0.05 (exact data not available)
Safety	All AEs, n (%) BEL/PBO, BEL/RTX, BEL/ST: 63 (87.5), 127 (88.2), 62 (81.6) Serious AE -infections-, n (%) BEL/PBO, BEL/RTX, BEL/ST: 2 (2.8), 8 (5.6), 1 (1.3)

Ab: antibodies. ACR: American College of Rheumatology. AE: adverse event. ANA: antinuclear antibodies. BEL: belimumab. BL: baseline. CI: confidence interval. CNS: central nervous system. CYC: cyclophosphamide. IV: intravenous. OR: odds ratio. PBO: placebo. Pts: patients. QW: once a week. RTX: rituximab. Sc: subcutaneous. SLE: systemic lupus erythematosus. SLEDAI-2K: Systemic Lupus Erythematosus Disease Activity Index -2000. ST: standard treatment. W: week.

## Data Availability

Data sharing is not applicable to this article.
